# Chronic Inflammatory Demyelinating Polyradiculoneuropathy and Diabetes: A Case Report

**DOI:** 10.7759/cureus.29390

**Published:** 2022-09-21

**Authors:** Inês Ferraz de Oliveira, Iuri Correia, Joana Urzal, Simão Cruz, Fernando Aldomiro

**Affiliations:** 1 Internal Medicine, Hospital Professor Doutor Fernando Fonseca, Amadora, PRT; 2 Neurology, Hospital Professor Doutor Fernando Fonseca, Amadora, PRT

**Keywords:** diabetic microvascular complications, diabetic neuropathy, distal symmetric polyneuropathy, diabetes mellitus, chronic inflammatory demyelinating polyradiculopathy

## Abstract

We present a case of a 42-year-old female living with poorly controlled diabetes who presented with a nine-month evolution of ataxic gait, reduced motor and sensitive function of lower and upper limbs, and postural anesthesia of fingers, feet, and toes. Deep tendon reflexes were abolished in the lower limbs and markedly diminished in the upper limbs. Cerebrospinal fluid (CSF) analysis showed a high protein level, and both imaging and serologic studies were normal. Although she had a previous electrophysiologic study showing distal symmetric polyneuropathy (DSPN) with an axonal lesion, nerve conduction studies were repeated, and a diagnosis of chronic inflammatory demyelinating polyneuroradiculopathy (CIDP) was made. According to the state of the art, intravenous immunoglobulin (IVIg) was started. The patient’s Inflammatory Neuropathy Cause and Treatment (INCAT) score and Medical Research Council (MRC) Sum Score both improved after two cycles. Unfortunately, symptoms quickly recurred, and corticosteroids were introduced to try to delay symptom recurrence, although it worsened diabetes control. Later, IVIg was stopped due to nephrotic syndrome, and immunosuppression was initiated. CIDP is a potentially treatable disease, but the diagnosis must be made as soon as possible to start therapy and reduce sequelae. Neuropathy in patients living with diabetes is common, but patients must be monitored closely to enable a correct diagnosis and adequate treatment.

## Introduction

Chronic inflammatory demyelinating polyradiculoneuropathy (CIDP) is an acquired immune-mediated disorder of peripheral nerves [1]. Although there is no clear correlation with any disease, patients frequently have one or more comorbidities, with hypertension, diabetes mellitus (DM), and other immune diseases being the most prevalent [2]. Diabetic neuropathies are the most prevalent chronic complications of diabetes and can affect different areas of the peripheral nervous system [3]. The presence of other diabetic neuropathies, such as distal symmetric polyneuropathy (DSPN), may prove challenging for CIDP diagnosis. The case we present examines a diagnosis of CIDP in a patient living with diabetes and DSPN.

## Case presentation

A 42-year-old female presented to the outpatient internal medicine clinic with a nine-month history of progressive hypoesthesia and muscle weakness of the upper and lower limbs progressing from distal to proximal regions and ataxic gait. She had been living with poorly controlled diabetes with microvascular complications (diabetic proliferative retinopathy, distal symmetric polyneuropathy, and microalbuminuria) and controlled hypertension. She was medicated with detemir insulin, metformin, vildagliptin, and enalapril. Family history was not relevant, with no history of neurologic disease.

Since the onset of symptoms, she had been observed at the emergency department for a vertigo episode where a cerebral computed tomography (CT) revealed no signs of ischemic nor hemorrhagic vascular lesion and no cerebral mass. Benign paroxysmal positional vertigo (BPPV) was excluded, and cervical CT was normal. All examination results are presented in Table 1.

**Table 1:** Examination results TSH: thyroid-stimulating hormone; FT3: free triiodothyronine; FT4: free triiodothyronine; HIV: human immunodeficiency virus; VDRL: Venereal Disease Research Laboratory; anti-SSA: anti-Sjögren’s syndrome A antibody; anti-SSB: anti-Sjögren’s syndrome B antibody; anti-Sm: anti-Smith; anti-RNP: anti-ribonucleoprotein; anti-dsDNA: anti-double-stranded deoxyribonucleic acid; anti-nRNP: anti-nuclear ribonucleoprotein; anti-PM-SCL: anti-polymyositis-scleroderma; PNMA2: paraneoplastic antigen Ma2; SOX1: SRY-box transcription factor 1; CT: computed tomography; MRI: magnetic resonance imaging; g/dL: grams per deciliter; mcL: microliter; pg/mL: picogram per milliliter; mIU/L: milli-international units per liter; ng/dL: nanogram per deciliter

Examinations	Results	Reference values
Blood		
Hemoglobin (g/dL)	12.2	11.5-16.5
White blood count (×10^12^/mcL)	7.3	4-11
Glycated hemoglobin (%)	12.5	3.8-5.8
Vitamin B12 (pg/mL)	>1000	211-911
Protein electrophoresis and immunofixation	Normal	-
TSH (mIU/L)	2.423	0.55-4.78
FT3 (pg/mL)	2.51	2.3-4.2
FT4 (ng/dL)	1.02	0.8-1.76
Antibodies anti-HIV1 and 2	Negative	-
VDRL	Negative	-
Systemic antibodies anti-SSA, anti-SSB, anti-Sm, anti-RNP, anti-dsDNA, anti-histones, anti-nRNP, anti-PM-SCL, anti-GAD2	Negative	-
Neurologic antibodies (calcium channels, Hu, Yo, Ri, CV2, PNMA2, anffins, recov, SOX1, titina)	Negative	-
Cerebrospinal fluid		
Cells	1	-
Glucose (mg/dL)	144	40-70
Proteins (mg/dL)	84.6	15-45
Tibbling index	0.54	0-10
*Borrelia* immunoglobulin M	Negative	-
Oligoclonal bands	Negative	-
Cerebral and cervical CT	Normal	-
Cerebral, cervical, and medullar MRI	Normal	-
Somatosensory evoked potential	Normal	-
Nerve conduction studies	Absence of sensory potential action of left sural and right median nerves; slight amplitude reduction of right radial nerve; marked motor potential action amplitude reduction of common peroneal nerves, left posterior tibial nerve, and right cubital nerve; prolonged distal latency of common peroneal nerves and right posterior tibial and median nerves; temporal dispersion in the proximal response of ulnar nerve; reduction of motor conduction velocity of common peroneal nerves and posterior tibial nerve; impersistence of F-waves in the inferior limbs; electrophysiologic study compatible with chronic demyelinating sensitive and motor polyneuropathy with axonal loss	-

Neurologic examination revealed no alteration of mental status or cranial nerves. She presented with an ataxic gait, being unable to walk without support or walk on her heels or toes. The Romberg test was positive, and minor terminal dysmetria was noted on the finger-nose test with a normal heel-knee test. Lower limb deep tendon reflexes were abolished and markedly diminished in the upper limbs. Motor function was altered with a positive Mingazzini test and diminished muscle strength with distal predominance being observed. Interosseous muscle atrophy was noted in both hands and feet. Sensory function examination of the upper limbs documented tactile hypoesthesia and hypoalgesia with postural hypoesthesia of the fingers. On the lower limbs, tactile anesthesia, analgesia, and vibratory hypoesthesia with postural anesthesia of feet and toes were present. Lhermitte’s sign was also present. At that point, posterior cord syndrome and peripheric neuropathy were considered.

Medullar and cerebral magnetic resonance (MRI) showed no traumatic abnormalities nor signs of vasculitis, and there was no history of radiation. Cerebrospinal fluid (CSF) showed hyperproteinorachia with albuminocytological dissociation. The Tibbling index was normal, and there were no oligoclonal bands. Somatosensory evoked potentials showed no alterations. Laboratory blood analysis confirmed a poorly controlled diabetes with glycated hemoglobin of 12.5%. Thyroid hormones were normal, and there was no vitamin B12 deficiency. Serologies for HIV, syphilis, *Borrelia* infection, and autoimmunity for neurologic and systemic diseases were negative. Protein electrophoresis and immunofixation were normal, excluding polyneuropathy, organomegaly, endocrinopathy, monoclonal protein, and skin change (POEMS) syndrome. Although there were previous nerve conduction studies (NCS) that showed axonal sensitive polyneuropathy, NCS was repeated due to new and rapidly progressive neurologic signs and symptoms. NCS revealed features of CIDP in more than two different nerves in both lower and upper limbs. At this point, a definitive typical CIDP diagnosis was confirmed, meeting all clinical criteria, electrophysiologic criteria in more than two nerves, and with cerebrospinal fluid protein elevation as supportive criteria.

According to the European Academy of Neurology/Peripheral Nerve Society (EFNS/PNS) guidelines, intravenous human immunoglobulin (IVIg) was started at a dose of 2 g/kg for five days with significant clinical improvement. Intensive glycemic control and neuromodulator pain therapy had already been implemented.

The patient was discharged after completing the first IVIg and was re-evaluated two weeks later. Due to significant clinical improvement after the first cycle of IVIg and after consulting with the neurology department, the same dose was repeated one month later. After the first two IVIg cycles, the Inflammatory Neuropathy Cause and Treatment (INCAT) disability scale score went from 6 to 3, and the Medical Research Council (MRC) Sum Score went from 42 to 54, a satisfactory response to the IVIg and another supportive criterion of CIDP diagnosis. Treatment was continued, according to EFNS/PNS guidelines, with a scheme of IVIg 1 g/kg for two days every three weeks.

Symptoms recurred soon after each infusion despite good glycemic control. After the fourth cycle of IVIg, glucocorticoids were introduced to control neurologic symptoms between IVIg infusions. Unfortunately, the patient developed nephrotic syndrome, and IVIg had to be suspended. Immunosuppression was started with azathioprine with very little result. Treatment of neuropathic pain was optimized, improving the patient’s quality of life.

## Discussion

Diagnostic criteria were reviewed in 2021, and CIDP is now classified as typical CIDP and CIDP variants [4]. Typical CIDP is characterized by a progressive or relapsing, symmetrical, proximal, and distal muscle weakness of upper and lower limbs, and sensory involvement of at least two limbs developing over at least eight weeks with absent or reduced tendon reflexes in all limbs. CIDP variants (distal, multifocal, focal, motor, or sensory) are classified according to nerve involvement and presentation [4].

Diagnosis may be challenging, and CIDP is often underdiagnosed or misdiagnosed as presentation varies widely and diagnostic criteria are only clinical and electrophysiologic [5,6]. Other diagnostic tests (cerebrospinal fluid analysis, magnetic resonance imaging, nerve biopsy, and somatosensory evoked potentials) can be performed, although none have yet been made part of guidelines due to their limited evidence in CIDP [6]. Additionally, acute presentation can occur, and patients may initially be diagnosed with Guillain-Barré syndrome (GBS) with later reclassification when symptoms continue to progress after eight weeks or relapse twice.

A retrospective study with 60 patients with definitive diagnoses of CIDP in a reference center showed that 68.3% had an alternative diagnosis, the most common being GBS (23.3%), often misdiagnosed in acute CIDP onset. Non-GBS alternative diagnoses (45%) were mainly misdiagnosed as genetic neuropathy, diabetic neuropathy, and chronic idiopathic axonal polyneuropathy [5].

Patients with CIDP often have comorbidities that may influence diagnosis and treatment choice, such as diabetes mellitus [2]. Although there is still controversy as to whether diabetes is a risk factor for CIDP, diabetes prevalence has been reported to be higher in patients with CIDP, compared with the general population [2,7,8].

Distal symmetric polyneuropathy (DSPN) is the most common diabetic neuropathy [3]. It presents with pain and dysesthesia when only small fiber function is impaired and loss of sensation when large fiber function is compromised. In some cases, DSPN might have demyelination and axonal loss, mimicking symptoms and sharing electrophysiologic findings of CIDP and therefore may influence CIDP diagnosis. For this reason, updated diagnostic criteria for CIDP for people that live with diabetes have been proposed [9] but are yet to be included in formal EFNS/PNS guidelines reviewed in 2021.

The first-line recommended treatment for CIDP is immunoglobulin (intravenous (IVIg) for induction and subcutaneous for maintenance), corticosteroids, or plasma exchange. EFNS/PNS guidelines advocate the first two as equally effective and recommend IVIg over plasma exchange due to ease of administration. Although there are no specific recommendations for CIDP treatment in people with diabetes, poor glycemic control in these patients is very common and usually results in IVIg use over corticosteroids [4].

Response rates to CIDP treatment seem to be similar in patients with and without diabetes, although underlying DSPN will limit the response, and full normalization of function and strength might not occur in patients with diabetes [10].

## Conclusions

Although the CIDP-DM association has yet to be clarified, people who live with diabetes, particularly patients with diabetic neuropathy, should be monitored closely. In these patients, previous neurologic symptoms may be a confounding bias, resulting in delayed CIDP diagnosis and consequently worse outcomes.

CIDP treatment should be implemented as soon as possible, alongside glycemic control and nerve pain control, to reduce symptoms and improve patients’ quality of life.

## Appendices

The diagnostic criteria for typical CIDP are shown in Table 2. 

**Table 2:** Diagnostic criteria for typical CIDP CIDP: chronic inflammatory demyelinating polyneuropathy; ULN: upper limit of normal values; LLN: lower limit of normal values; CMAP: compound muscle action potential; SNAP: sensory nerve action potential; MRI: magnetic resonance imaging; CSF: cerebrospinal fluid

Criteria	Indication
Clinical criteria (all three must be present)	Progressive or relapsing, symmetric, proximal, and distal muscle weakness of upper and lower limbs, and sensory involvement of at least two limbs
	Developing over at least eight weeks
	Absent or reduced tendon reflexes in all limbs
Motor nerve conduction criteria for demyelination (at least one must be present)	Motor distal latency prolongation ≥50% above ULN in two nerves (excluding median neuropathy at the wrist from carpal tunnel syndrome)
	Reduction of motor conduction velocity ≥30% below LLN in two nerves
	Prolongation of F-wave latency ≥20% above ULN in two nerves (≥50% if amplitude of distal negative peak CMAP <80% of LLN)
	Absence of F-waves in two nerves (if these nerves have distal negative peak CMAP amplitudes ≥20% of LLN) + ≥1 other demyelinating parameter in ≥1 other nerve
	Motor conduction block: ≥30% reduction of the proximal relative to distal negative peak CMAP amplitude, excluding the tibial nerve, and distal negative peak CMAP amplitude ≥20% of LLN in two nerves, or in one nerve + ≥ 1 other demyelinating parameter except absence of F-waves in ≥1 other nerve
	Abnormal temporal dispersion: >30% duration increase between the proximal and distal negative peak CMAP (at least 100% in the tibial nerve) in ≥2 nerves
	Distal CMAP duration (interval between onset of the first negative peak and return to baseline of the last negative peak) prolongation in ≥1 nerve + ≥1 other demyelinating parameter in ≥1
Sensory nerve conduction criteria (must be present)	Sensory conduction abnormalities (prolonged distal latency, or reduced SNAP amplitude, or slowed conduction velocity outside of normal limits) in two nerves
Supportive criteria (may support the diagnosis of CIDP in patients who fulfil clinical criteria for CIDP but whose electrodiagnostic criteria are only partially present)	Response to treatment (disability and impairment score scale improvement)
	Ultrasound (nerve enlargement of at least two sites in proximal median nerve segments and/or the brachial plexus)
	MRI (enlargement and/or increased signal intensity of nerve root(s) on T2-weighted MRI sequences)
	CSF analysis (CFS protein elevation)
	Nerve biopsy (thinly myelinated axons and small onion bulbs, and/or thinly myelinated or demyelinated internodes in teased fibers, and/or perivascular macrophage clusters, and/or supportive features of demyelination on electron microscopy)
